# Monolithic Microwave-Microfluidic Sensors Made with Low Temperature Co-Fired Ceramic (LTCC) Technology

**DOI:** 10.3390/s19030577

**Published:** 2019-01-30

**Authors:** Karol Malecha, Laura Jasińska, Anna Grytsko, Kamila Drzozga, Piotr Słobodzian, Joanna Cabaj

**Affiliations:** 1 Faculty of Microsystem Electronics and Photonics, Wrocław University of Science and Technology, Wybrzeże Wyspiańskiego 27, 50-370 Wrocław, Poland; laura.jasinska@pwr.edu.pl; 2 Faculty of Electronics, Wrocław University of Science and Technology, Wybrzeże Wyspiańskiego 27, 50-370 Wrocław, Poland; anna.grytsko@pwr.edu.pl (A.G.); piotr.slobodzian@pwr.edu.pl (P.S.); 3 Faculty of Chemistry, Wrocław University of Science and Technology, Wybrzeże Wyspiańskiego 27, 50-370 Wrocław, Poland; kamila.drzozga@pwr.edu.pl (K.D.); joanna.cabaj@pwr.edu.pl (J.C.)

**Keywords:** microwaves, microfluidic, sensor, LTCC

## Abstract

This paper compares two types of microfluidic sensors that are designed for operation in ISM (Industrial, Scientific, Medical) bands at microwave frequencies of 2.45 GHz and 5.8 GHz. In the case of the first sensor, the principle of operation is based on the resonance phenomenon in a microwave circuit filled with a test sample. The second sensor is based on the interferometric principle and makes use of the superposition of two coherent microwave signals, where only one goes through a test sample. Both sensors are monolithic structures fabricated using low temperature co-fired ceramics (LTCCs). The LTCC-based microwave-microfluidic sensor properties are examined and compared by measuring their responses for various concentrations of two types of test fluids: one is a mixture of water/ethanol, and the other is dopamine dissolved in a buffer solution. The experiments show a linear response for the LTCC-based microwave-microfluidic sensors as a function of the concentration of the components in both test fluids.

## 1. Introduction

Recently, there has been a noticeable increase in interest in integrated microfluidic systems. These miniature devices can handle very low volumes of fluid, which allows them to operate using smaller amounts of reagents and produce less waste in comparison to classical analytical equipment. Moreover, their smaller size makes these devices portable. As a result, it is possible to carry out analyses both in laboratory and field conditions. A typical microfluidic system consists of several functional blocks. Micropumps and microvalves are responsible for the transportation of a fluid sample, micromixers for the preliminary preparation of this sample, the microreactor for carrying out appropriate (bio)chemical reactions, and the detection system for determining the content of the products of the performed reaction. Usually, microfluidic systems use the optical or electrochemical principle to determine the analyte [1,2,3]. These kinds of detection methods are characterized by high selectivity and sensitivity, but in most cases, such methods do not allow us to measure the analyte concentration directly. Very often, before the measurement, it is necessary to carry out an analytical reaction, which requires the use of additional reagents or a catalyst (e.g., enzyme). The enzyme immobilization process inside the microfluidic system is usually complicated and time-consuming [4,5,6]. The microwave-microfluidic systems appear to be a very attractive alternative. Among different applications of such devices, there are three that are used more commonly. The first application is related to the microwave heating of liquids. This technique makes it possible to selectively deliver energy to an analyte without affecting the surrounding materials [7,8,9,10,11]. The second application consists of the enhancement of chemical reactions, e.g., the synthesis of nanoparticles [12]. The last domain of application is the characterization of the electrical parameters of various (bio)chemical substances that flow through a microchannel [13,14,15]. This last method can also be used in the process of characterization of various (bio)chemical compounds. One of the methods of detecting physical or chemical changes within a microfluidic system is based on microwave resonance. Withayachumnankul et al. [16] developed a microwave-microfluidic sensor for the dielectric characterization of fluids. It was based on a split-ring resonator (SRR) coupled to a microstrip line. The sensor was made with PCB (printed circuit board) technology. The SSR, microstrip and ground plane were made of gold-coated copper. The high-frequency laminate (ceramic-Poli(tetrafluoroetylen) composite) was used as a substrate. The microfluidic part of the sensor was made of PET (polyethylene terephthalate) film, which was mechanically pressed against the substrate. The performance of the sensor was examined for several ethanol/water mixtures, and a linear response for various concentrations of the test fluid was obtained for this construction. A slightly different construction was proposed by Abduljabar and co-workers [17]. They fabricated a two-gap split resonator using microstrip technology. The PCB laminate (RT/duroid 5880, Rogers Corp., Chandler, AZ, USA) was used as a substrate and the metallization was made of copper. The investigated fluid was introduced into the resonator by the quartz or PFA (perfluoroalkoxy alkanes) capillary, which was mechanically fixed inside the gap regions. Several chemical substances were used to test the sensing properties of the resonator. The performed experiments showed that the device was sensitive to small changes in the permittivity of the fluids. This provided a means of fluid identification. The microfluidic system for microwave sensing and heating was proposed by Boybay et al. [18,19]. The electrical part of the device consisted of a circular inner loop (resonator) and an outer excitation loop. The inner loop had a small gap through which a microchannel was aligned. The signal lines were deposited on a glass substrate using a combination of photolithography and electroplating. The microfluidic part of the device was made of PDMS (polydimethylsiloxane). Both parts were bonded together using oxygen plasma. The performed measurements showed that the sensor was able to selectively heat and detect various fluids. Another method that can be used in the identification of (bio)chemical fluids by the measurement of their dielectric properties was proposed by Song and Wang [20]. They developed a microwave-microfluidic system which consisted of a Wilkinson power divider and a rat-race hybrid. The input power was split into two signal lines. A microchannel was placed above each line. When both microchannels were filled with the same fluid, the signals at the output of the rat-race hybrid canceled out. For microchannels filled with the reference and test fluids, a non-zero microwave signal appeared at the output. The resulting output signal was proportional to the concentration of the test fluid. Microwave components of that device were deposited on a glass substrate and microchannels were made in PDMS. Both materials were bonded using oxygen plasma. A similar system was developed using PCB technology [21].

Until recently, microfluidic-microwave systems were mainly made using PCB and polymer technologies. However, both techniques have some limitations. In the case of PCB technology, the main issues are fabrication and the integration of microfluidic structures, while the main problem with polymer technology is the integration of electronic components. This issue can be overcome using glass substrates, on which conductive layers can be deposited using thin-film technology or photolithography before bonding with the polymer. However, this makes the technological process more complicated, expensive and time-consuming. One solution for all the aforementioned problems can be the use of LTCC (low temperature co-fired ceramic) technology. An advantage of using LTCC technology in comparison with PCB and polymer technologies is the possibility to integrate microfluidic and electronic components in a monolithic ceramic substrate [22,23]. Microfluidic structures can be easily fabricated using laser micromachining, mechanical milling or hot embossing [24,25,26]. Details about microchannel fabrication process using multilayer LTCC technology can be found in [27]. Electrical paths made of highly conductive metals and alloys (e.g., Cu, Ag, Au, Pt, AgPd) can be deposited onto a ceramic substrate using screen-printing or ink-jet printing methods. Moreover, the LTCC material is chemically resistant, inert, stable in high pressures and temperatures, and has very good electrical properties at high frequencies (with a stable dielectric constant and low loss tangent up to 60 GHz). The dielectric losses have an influence on quality factor of the resonator. The lower the loss tangent, the higher the quality factor. The sensitivity of the resonator-based sensor strongly depends on the quality factor, therefore the LTCC is a very good choice as a substrate material for such devices. However, according to our best knowledge, there is no publication on application of LTCC for fabrication of microwave-microfluidic sensors.

The goal of the research presented in this paper is an attempt to apply the LTCC technology for fabrication of monolithic microfluidic-microwave sensors. To achieve this goal two types of LTCC-based sensors were designed and fabricated. Moreover, their sensing properties were experimentally examined and compared. Both devices used different methods of detecting the substance that flowed through the microchannel. In the case of the first sensor, the principle of operation was based on the measurement of the response of the microwave resonance circuit (a resonator). The second sensor was based on the interferometric principle that was implemented with the use of a microwave ring coupler. In this case, the response (output signal) was the result of the superposition of two signals that are transmitted through a channel with a test sample and a reference channel, respectively. Both sensors were tested by means of two different fluids: an ethanol/water mixture and a solution of dopamine in a buffer.

## 2. Sensor Construction and Principle of Operation

### 2.1. Resonator-Based Sensor

**Figure 1:**
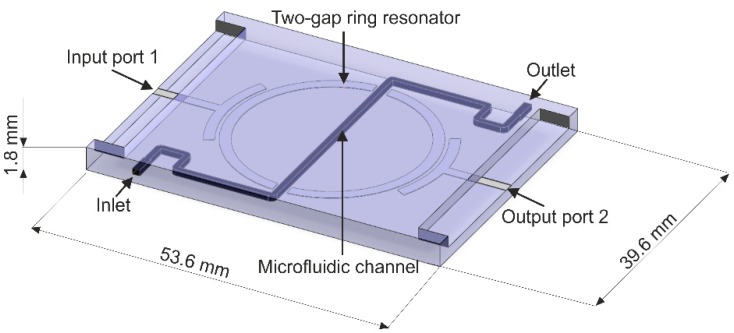
A computer-aided design (CAD) model of the two-gap ring resonator sensor.

A computer-aided design (CAD) model of the resonator-based sensor is presented in Figure 1. The structure consists of a microstrip circuit printed on a dielectric layer and a microfluidic channel. The input and output port of the circuit is connected to microstrip lines, which are capacitively coupled to the two-gap ring resonator. The microwave structure is covered with an additional dielectric layer and the microfluidic channel is placed between them, and runs through the gaps of the resonator.

The microwave signal is coupled via the input feed line to the ring resonator. At the resonant frequency, the equivalent circuit model of this structure can be approximated by a simple resonant LC circuit. It consists of an inductance (L), formed by two half loops of ring resonator, connected in series with a capacitance (C) that is introduced by the two gaps. The capacitance of the gaps depends on the permittivity of the fluid that flows through the microchannel. As a result, any change that occurs in the fluid composition is observed as a change in the resonant frequency of the structure, which, in turn, causes changes in the amplitude of the output signal. The sensitivity of the sensor depends on the quality factor (Q) of the resonator. The higher the quality factor, the higher the sensitivity. High-quality factors require the use of low-loss materials, so the LTCC would be a very good choice. The geometry of the resonator was designed by means of CAD tools. The modeling and microwave analysis were performed using COMSOL Multiphysics software. In order to reduce the number of unknowns and to decrease calculation time, the microstrip structure was assumed to be of zero thickness and infinite conductivity (a perfect electric conductor (PEC) was used for all metal surfaces). The parameters of the modeled water and LTCC materials are presented in Table 1. 

**Figure 2:**
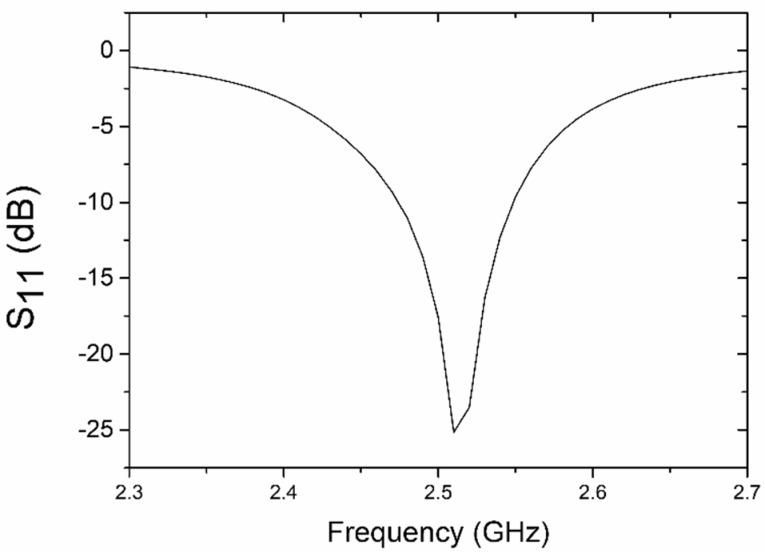
Simulated reflection coefficient of the resonator-based sensor (microchannel filled with deionized water).

The microchannel was modeled as a three-dimensional dielectric body embedded in a finite-size dielectric substrate. The result of the electromagnetic simulations of the reflection coefficient (S_11_) in the input port of this sensor is presented in Figure 2. As we can see, the resonant frequency was tuned to 2.51 GHz under the condition of a microchannel loaded with deionized water (the load was modeled by assigning the dielectric constant of water to the body of the microchannel). The presence of a distinct minimum reflection coefficient (−25 dB) close to 2.5 GHz indicates that the input impedance of the resonator is well-matched to the source of a microwave signal. The same pertains to the output port (the structure is electrically symmetric). Since the resonator is a very low loss passive circuit, and deionized water introduces no loss, we can conclude that almost all microwave power is transmitted from the input to the output port of this structure at resonance. The change in the dielectric constant of the microchannel detunes the resonator, so the signal transmitted at the original resonant frequency will start to reflect at the input port of the sensor. This, in turn, will decrease the amplitude of the signal at the output port of this sensor. In this way, we obtain information about any changes in the dielectric properties of the (bio)chemical compound that flows through the microchannel. It is also worth mentioning that the operating point of the sensor can be put in the slope of the S_11_ curve (by selecting a lower or higher frequency), so the output signal can increase or decrease, showing the direction of the changes in the test fluid.

### 2.2. Coupler-Based Sensor

**Figure 3:**
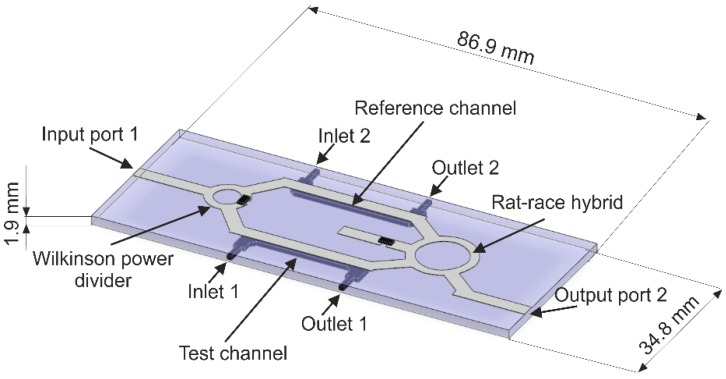
A CAD model of the coupler-based sensor.

The construction of the sensor was based on [20]. Its CAD model is presented in Figure 3. The structure contains a few connected microstrip elements, which consists of input and output microstrip lines, Wilkinson’s 3-dB power divider, the rat-race hybrid (the ring coupler) and two microstrip lines (reference and test) in between. We can also see two microchannels, with coupled inlets and outlets. The microwave circuit is printed on a dielectric substrate and the two microchannels are embedded in the substrate just beneath the reference and the test line, respectively.

The principle of operation is as follows: one microchannel is filled with a reference sample and the second one is filled with a test sample. The input signal is transmitted from port 1 to the 3-dB power divider that divides the signal equally in two. The resulting signals are then transmitted simultaneously through two separate microstrip lines to input ports of the rat-race hybrid. The microchannels with fluid samples placed beneath those lines have a strong influence on the electrical length of those lines, and this enables different phase shifts on each line. By introducing different samples into the test and reference channel, a phase difference between the transmitted signals is produced. These signals enter the rat-race hybrid and superimpose (interfere with) in its output port, so that the output signal is proportional to the phase difference. The principle of operation for the rat-race hybrid is such that two input signals of the same phase will cancel each other out, so the resulting output signal is zero. On the other hand, the response of the hybrid will reach its maximum when the input signals are out-of-phase. Therefore, for microchannels filled with the same fluid, the output signal is zero. In turn, when the microchannels are filled with two different fluids that have different electric and magnetic constants, this will result in a non-zero output signal. This signal can be used for detecting changes in the parameters and/or properties of fluids that are used in the test.

**Figure 4:**
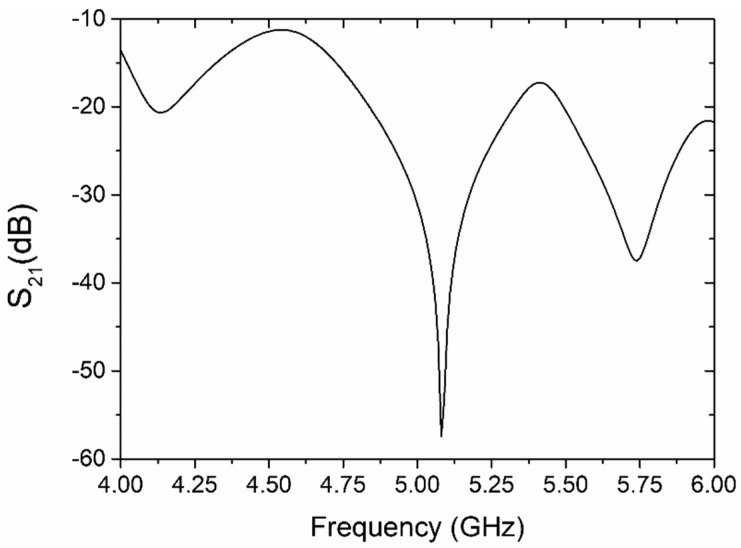
Simulated transmission coefficient of the coupler-based sensor (reference and test channels filled with deionized water).

The sensor was designed using electromagnetic numerical simulations. The numerical modeling and analysis were performed by means of the COMSOL Multiphysics software. We modeled the structure of the coupler-based sensor based on the same assumptions as in the case of the resonator-based sensor. The operating frequency (*f_0_*) of this sensor was tuned to ca. 5 GHz under the condition of reference and test channels that were filled with the same fluid (deionized water). In Figure 4, we can see the simulated transmission coefficient (S_21_) between the input and output port of the sensor (a ratio of the output and input power). The deep minimum in the vicinity of 5 GHz (−57.3 dB at *f_0_*) means that there is an almost ideal cancelation of the interfering signals, and this means that both channels have been filled with the same fluid. Now, when the properties of the fluid inside the test channel are changed, a non-zero signal at *f_0_* is produced at the output port. This effect can also be observed as a shift of the minimum in S_21_ to the right or left, provided the impedance of the test line does not change much. The sensitivity of this type of sensor depends on several factors: the quality of impedance matching at the input and output ports of the microwave circuit, the accuracy of phase balance between the reference and test lines, and the isolation of both the power divider and the ring coupler. All of these requirements are strongly related to the accuracy of the technology that will be used in the fabrication of the sensor.

## 3. Technology

The resonator-based and coupler-based microwave-microfluidic sensors were made with LTCC technology. The DuPont 951 Green Tape system was used to make both devices. Conductive layers made of silver (DuPont LL612, DuPont, Wilmington, DE, USA) were deposited using a standard screen-printing method (Aurel VS 1502A) through a 325-mesh steel screen. The registration orifices, vias, microchannels and other shapes were cut in a green LTCC material using a UV laser (LPKF Protolaser U, LPKF Laser & Electronics, Garbsen, Germany). After laser micromachining and screen-printing, the LTCC tapes were stacked and laminated using an isostatic press. The resulting laminates were co-fired in a box furnace (Naberthern L3/S, Lilienthal, Germany). The process was performed in air with a maximum temperature of 875 °C, according to the standard thermal profile recommended by DuPont.

### 3.1. Resonator-Based Sensor

**Figure 5:**
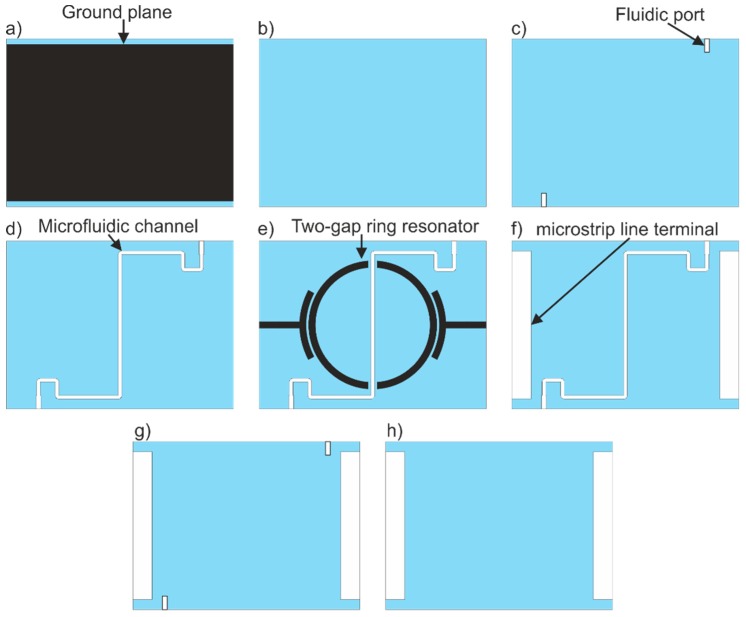
The LTCC layers for the resonator-based sensor: (**a**) bottom layer with deposited ground plane, (**b**) bottom layer, (**c**) layer with cuts for fluidic ports, (**d**) layer with microfluidic channels, (**e**) layer with deposited two-gap ring resonator, (**f**) layer with microchannel and microstrip terminal, (**g**) top layer with cuts for fluidic port, (**h**) top layer with cuts for microstrip terminals.

The device consisted of 12 LTCC layers. Each was 165-um thick before firing. The sensor layout is presented in Figure 5. Three layers define the bottom of the sensor (Figure 5a,b). The lowest layer (Figure 5a) was covered by a silver paste to form the ground plane. The 2 subsequent layers (4th and 5th) consist of cuts for fluidic ports (see the pattern in Figure 5c). The microfluidic channel makes up the next 3 layers (Figure 5d–f). The two-gap ring resonator and microstrip lines are printed on the middle layer, as shown in Figure 5e. The 9th and 10th layers contain cuts for microfluidic inlets and outlets, and for microstrip line ports (Figure 5g). The last two layers (Figure 5h) define the top of the sensor.

In order to prevent sagging of the microchannel and deformation of other spatial structures, a sequential lamination was performed. In the first step, the 3 bottom LTCC layers (Figure 5a,b) were laminated. At the same time, the 4 top layers (Figure 5g,h) were pressed. The lamination process of the bottom and top layers was performed at 20 MPa, 70 °C for 10 min. Next, the 3 layers containing the microchannel were laminated (5 MPa, 70 °C, 10 min). Finally, all 3 parts were pressed together (5 MPa, 70 °C, 10 min). The fluidic ports made of stainless steel pipes cut from medical needles (diameter 0.8 mm) were glued together using epoxy resin and the SMA (SubMiniature version A) connectors were soldered to the fired LTCC structure.

**Figure 6:**
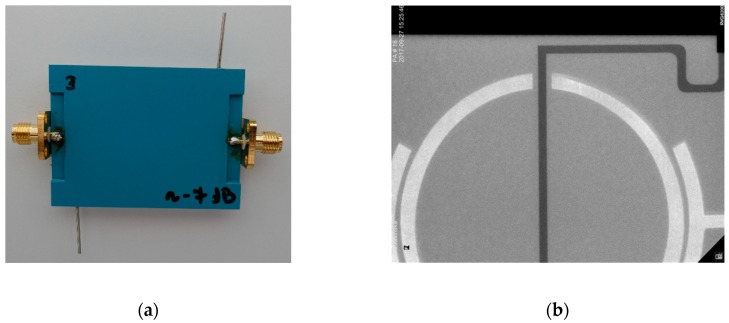
The resonator-based sensor: (**a**) photo, (**b**) X-ray image for a resonator and microchannel alignment.

The resonator-based sensor made with LTCC technology and an X-ray image of its fragment are presented in Figure 6. As can be seen in Figure 6b, the microchannel and resonator gap are properly aligned.

### 3.2. Coupler-Based Sensor

**Figure 7:**
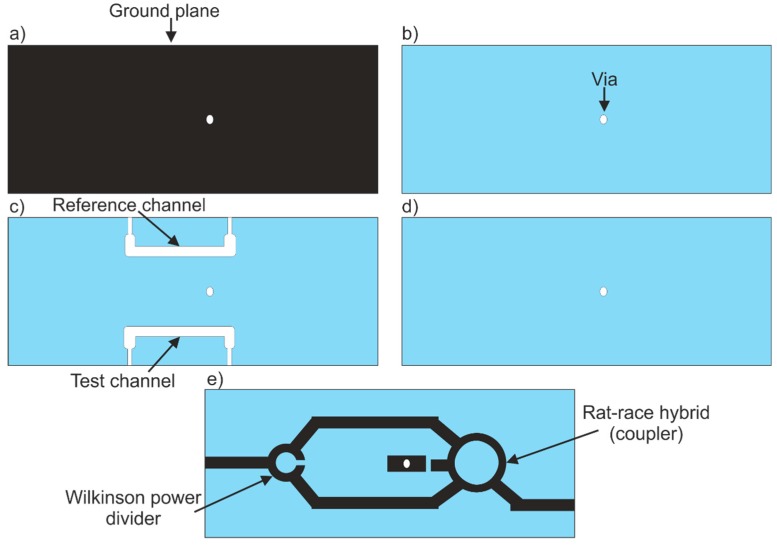
The LTCC layers for the coupler-based sensor: (**a**) bottom layer with deposited ground plane and via hole, (**b**) bottom layer with via hole, (**c**) middle layer with reference and test channels, (**d**) cover layer with via hole, (**e**) top layer with deposited microwave circuits.

Nine layers of a 254 µm-thick (before firing) LTCC material were used to make a coupler-based sensor. All the patterned layers are presented in Figure 7. The bottom of the sensor consisted of 3 layers (Figure 7a,b). The 1st was covered with a silver paste to form the ground plane (Figure 7a). The reference and test channels were cut into 3 middle layers (Figure 7c). The channels were sealed by 3 layers (Figure 7d,e). The microstrip lines, Wilkinson power divider and rat-race hybrid were deposited on the surface of the top layer (Figure 7e).

**Figure 8:**
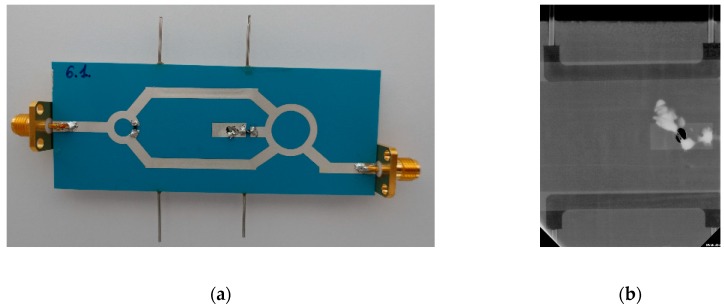
The coupler-based sensor: (**a**) photo, (**b**) X-ray image of microstrip lines and channel alignment.

In all LTCC layers, a via is cut for electrical interconnection between the rat-race hybrid and the ground plane. As previously mentioned, the lamination process was divided into a few steps. First, the 3 bottom layers were pressed (Figure 7a,b) while simultaneously, the 3 top layers were laminated (Figure 7d,e). The top and bottom layers were pressed with a pressure of 20 MPa, at 70 °C for 10 min. Next, the 3 layers containing the reference and test channels were laminated. In order to prevent channel deformation, the layers were pressed with lower pressure (5 MPa, 70 °C, 10 min). Finally, all parts were stacked and laminated (5 MPa, 70 °C, 10 min). After that, the LTCC laminate was co-fired, and fluidic ports (stainless steel pipes with a diameter of 0.8 mm) were glued using epoxy resin to the fired LTCC structures. In order to isolate two branches of the Wilkinson power divider, a 100-ohm SMD (surface mounting device) resistor was soldered. Another 50-ohm SMD resistor was used to terminate the port of the rat-race hybrid. The ready-to-use coupler-based sensor made of the LTCC is presented in Figure 8a. As can be seen in the X-ray image (Figure 8b), both channels are placed directly under the microstrip lines.

## 4. Experimental Verification and Discussion

**Figure 9:**
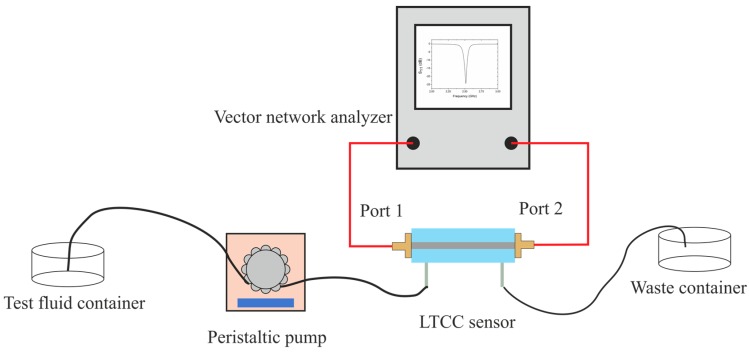
Scheme of the experimental test-bed used for testing the developed sensors.

The sensing properties of both LTCC-based microwave-microfluidic systems were verified experimentally. Measurements were performed by means of an experimental test-bed, shown in Figure 9. Two types of test fluid were used during the measurements: a water/ethanol mixture and dopamine dissolved in a phosphate-citrate buffer of pH 5.2. The volume fraction of water in ethanol was changed from 0% (pure ethanol, *ε_r_* = 25.3) to 100% (pure deionized water, *ε_r_* = 80.1). The concentration of dopamine varied from 0.1 to 200 mM. The microchannels of both sensors were filled with test fluids using a peristaltic pump (Ismatec Regolo ICC, Ismatec, Wertheim, Germany). During all experiments, the flow rate was set to 0.25 mL/min. Scattering parameters were measured by means of a vector network analyzer (VNA, Keysight N9923A, Keysight, Santa Rosa, CA, USA). The measurements were performed for frequency range from 2.3 to 2.7 GHz (for resonator-based sensor) and from 4 to 6 GHz (for coupler-based sensor) with step size between frequency points equal to 0.001 GHz. Before performing the measurements, the VNA was calibrated. All experiments were performed in an air-conditioned laboratory at room temperature.

### 4.1. Resonator-Based Sensor

**Figure 10:**
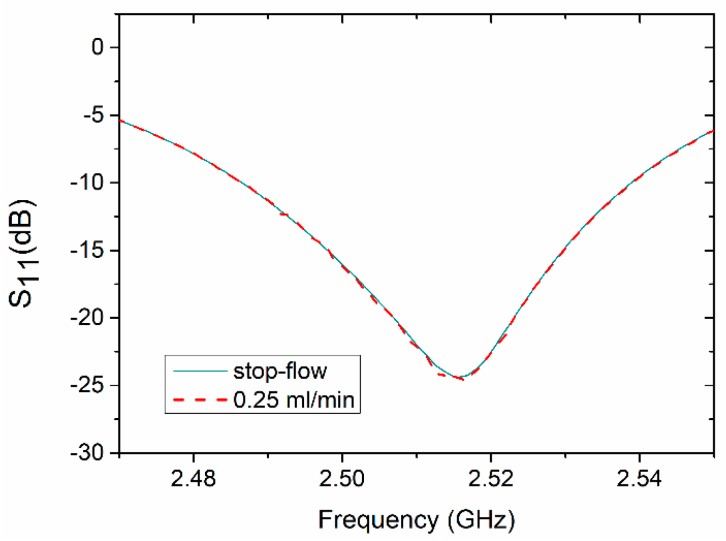
Reflection coefficient of the resonator-based sensor measured for stop-flow and continuous flow modes.

The reflection coefficient (S_11_) of the sensor was measured to detect the fluid in the microchannel. In order to determine the influence of the continuous flow on the results, the reflection coefficient was measured under two different conditions (modes). First, the microchannel was filled with deionized water and the flow was stopped during measurement (stop-flow mode). Then, the measurements were performed with a continuous flow (0.25 mL/min) of deionized water. As we can see in Figure 10, there is no significant difference between both measured reflection characteristics. The difference is negligibly smaller than 0.005 dB. Moreover, the resonance frequency was the same in both cases. 

**Figure 11:**
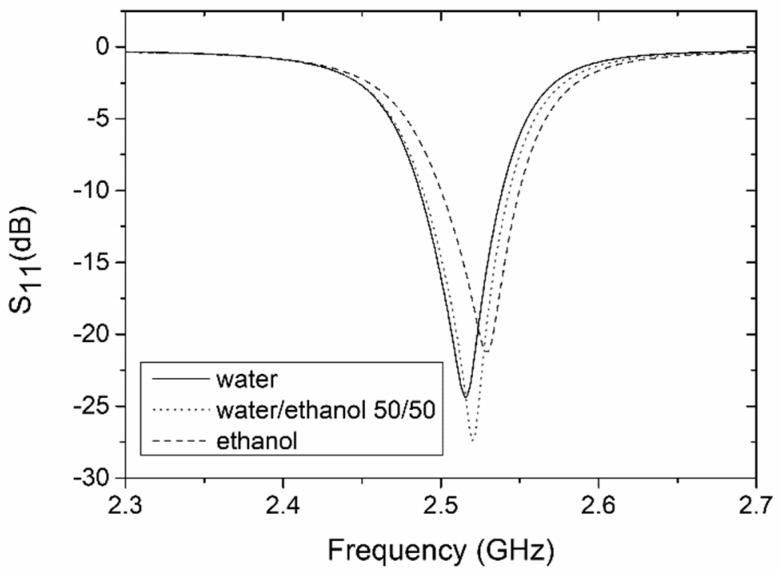
Measured reflection coefficients of the resonator-based sensor for a microchannel filled with water, ethanol, and a 50/50 water/ethanol mixture.

The rest of the measurements were taken under a continuous flow. In order to detect changes in the test fluid, we decided to observe the shift in the sensor’s resonant frequency. The measured S_11_ for the deionized water, the 50/50 water/ethanol mixture, and the ethanol as a function of the frequency, is presented in Figure 11. The resonant frequency of the system filled with deionized water was 2.515 GHz, where a minimum reflection of −24.4 dB was measured. We can observe good agreement between the simulated (S_11_ = −25 dB at 2.51 GHz) and measured (S_11_ = −24.4 dB at 2.515 GHz) results when we compare Figure 2 and Figure 11.

**Figure 12:**
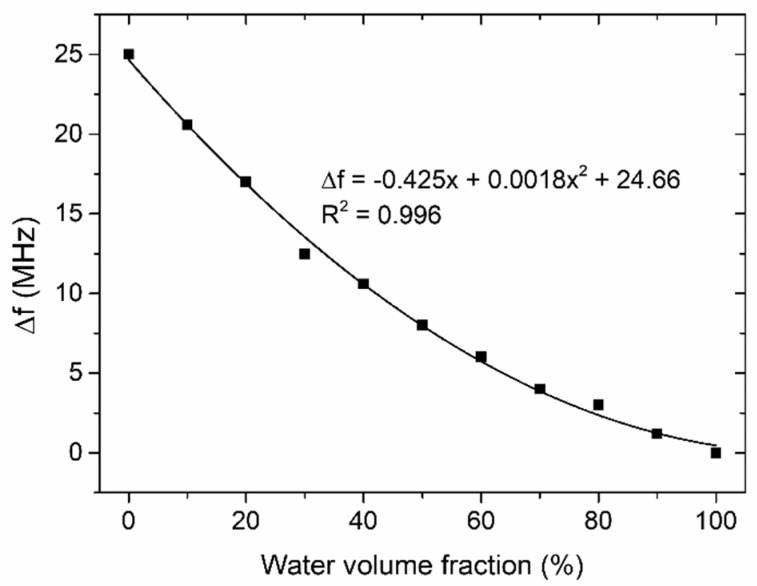
The resonance frequency variation as a function of the water volume fraction in a water/ethanol mixture.

Small differences in the values of the resonant frequency (~5 MHz) and amplitude of S_11_ (~0.6 dB) are found among the calculations (simulation) and measurements. This is probably caused by differences between the designed and real microwave components, due to inaccuracies introduced by the LTCC technology itself, e.g., the thickness of the silver conductor, the variation of the substrate permittivity due to the firing process [28], and the inaccuracy of the dimensions of the microstrip lines. As we can see, the maximum resonant frequency changes when the microchannel is filled with various mixtures of deionized water and ethanol. A shift of resonant frequency with respect to pure deionized water was found to be approximately 25 MHz for the 96% ethanol mix. The reflection peak of the resonant frequency moved from −24.4 dB for deionized water to −21.3 dB for the 96% ethanol mix. This is obviously a parasitic effect caused by the excessive coupling of the resonator to the input and output ports. In order to observe a shift in frequency only, the coupling of the resonator should be weak. The change of resonant frequency as a function of the volume fraction of deionized water is shown in Figure 12. As we can see, this change is proportional to the water volume fraction, and this relation can be approximated by means of a polynomial of the water volume fraction (x) with R^2^ = 0.996. 

**Figure 13:**
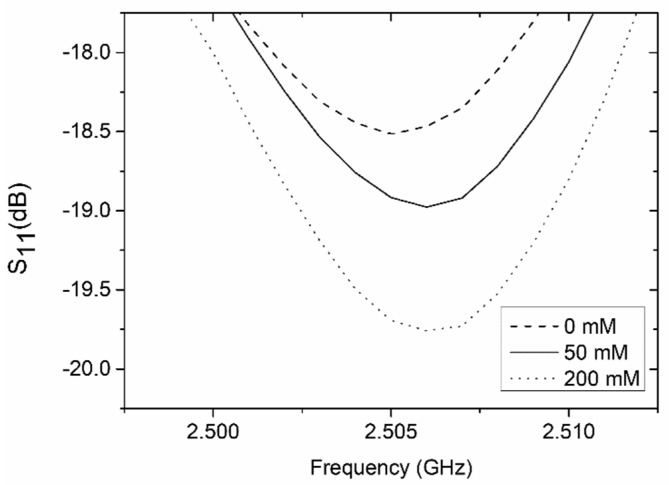
Measured reflection coefficient of the resonator-based sensor for a microchannel filled with buffer and dopamine (50 mM and 200 mM).

The resonator-based sensor was also applied to determine the concentration of dopamine in a liquid sample. Commonly, for the detection of dopamine, the electrochemical or optical method (fluorescence measurement) is used. However, both methods require the preparation of the analyte prior to taking measurements. For example, in the case of fluorescence measurements, the enzymatic oxidation of dopamine to dopamine-o-quinone, and then to polydopamine is carried out. This requires the immobilization of the enzyme (laccase) inside the microfluidic system. This process is both complicated and time-consuming [29]. To demonstrate the possibility of the direct detection of dopamine, the resonator-based sensor was used. The measured reflection coefficient of the sensor, for various concentrations of dopamine as a function of frequency, is presented in Figure 13. 

**Figure 14:**
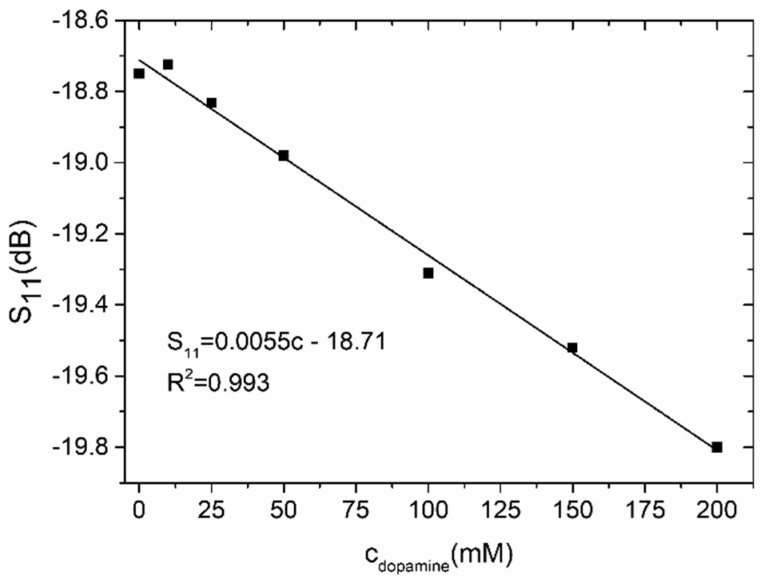
Reflection coefficient as a function of the dopamine concentration.

The resonant frequency for the microchannel filled with a pure buffer was ca. 2.5 GHz, and it did not change significantly (less than 1 MHz) when the microchannel was filled with dopamine concentrations. However, the reflection magnitude decreased with the increased concentration of dopamine. The magnitude of S_11_ changed from −18.5 dB (for pure buffer) to ±19.8 dB (for 200 mM of dopamine). The reflection magnitude as a function of the dopamine concentration is shown in Figure 14. Theoretically, it would be possible to measure a dopamine concentration of 50 mM. However, very low sensitivity (5.5 × 10^−3^ dB/mM) precludes the resonator-based sensor from the practical testing of the dopamine concentration. Based on the obtained results, it can be concluded that the presented LTCC resonator-based sensor is better suited to testing the composition of liquid samples.

### 4.2. Coupler-Based Sensor

**Figure 15:**
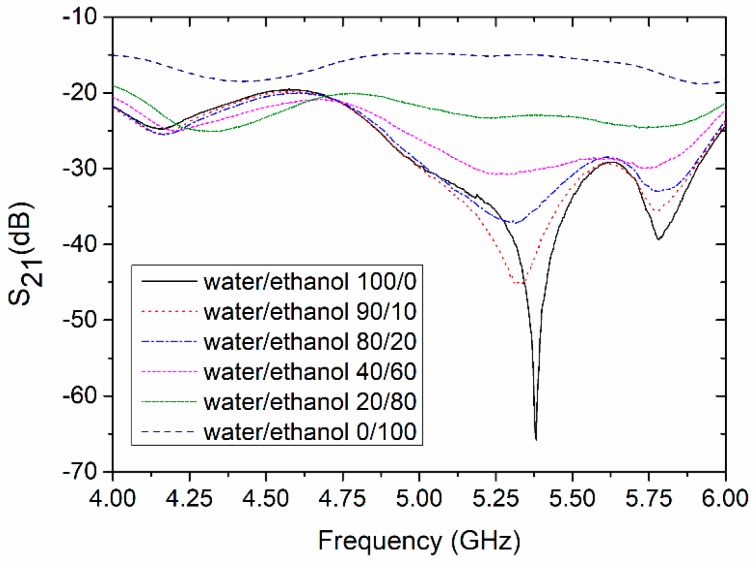
Measured transmission coefficient of the coupler-based sensor (reference channel filled with water).

In order to characterize the performance of the LTCC-based sensor, the transmission characteristic (S_21_) was measured using VNA. Measurements were performed for the reference channel filled with deionized water and the test channel filled with various volume fractions of deionized water in ethanol (from 10% to 90%). The results of the measurements are presented in Figure 15. As we can see, the magnitude of S_21_ for both channels filled with deionized water has minimal value at ca. 5.38 GHz. The value of S_21_ at resonant frequency was −65.8 dB. This means that the output power was more than two million times smaller than the input power. The characteristic frequency shifted by 0.28 GHz from the original design (5.1 GHz), probably due to differences in the designed and fabricated LTCC device (e.g., uncertainty in the permittivity of the substrate or the thickness of the conductor layer made with screen-printing). 

**Figure 16:**
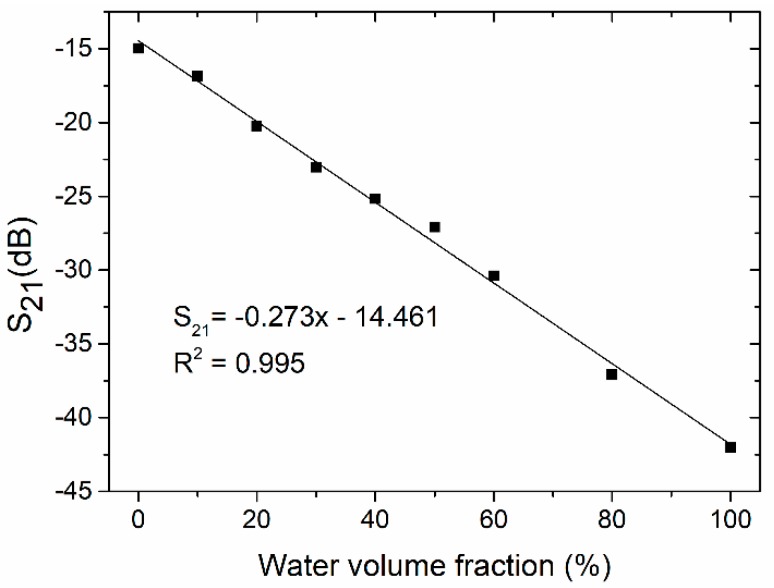
Transmission coefficient as a function of the water volume fraction in the water/ethanol mixture.

For the test channel filled with a mixture of water and ethanol, the operating frequency shifted to 5.33 GHz and the transmission magnitude decreased with the increase of ethanol in the mixture. The S_21_ parameter was used as a sensing indicator. Its value as a function of the volume fraction of deionized water in the water/ethanol mixture is shown in Figure 16.

As we can see, the decrease in magnitude of S_21_ was proportional to the water content in the test mixture. As a result, a linear relationship was achieved (R^2^ = 0.995). The slope of the S_21_ curve was equal to 0.273 dB per unit volume fraction of water.

**Figure 17:**
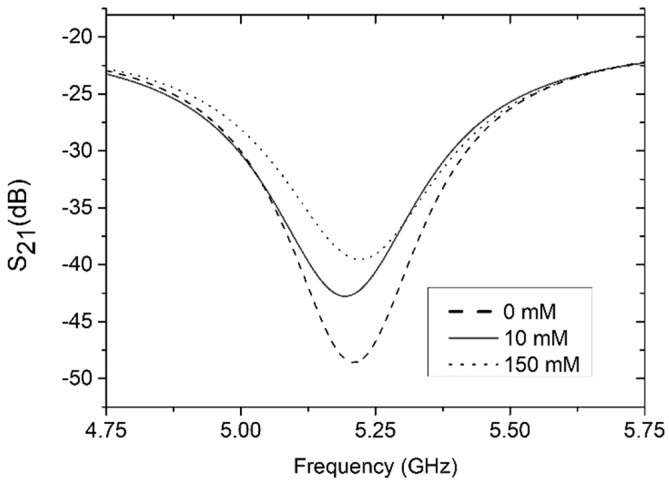
Measured transmission coefficient of the coupler-based sensor for various concentrations of dopamine (reference channel filled with buffer).

**Figure 18:**
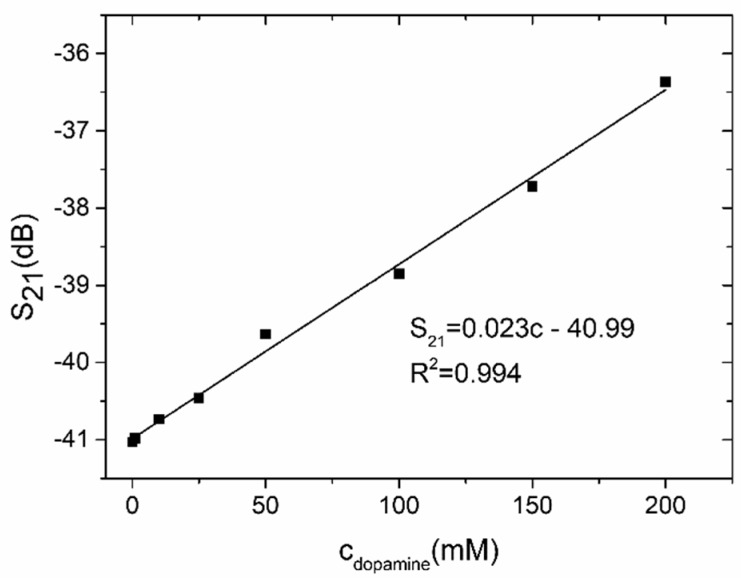
Transmission coefficient as a function of dopamine.

The second test was performed for the reference channel filled with a phosphate-citrate buffer and a test channel filled with various concentrations of dopamine dissolved in the buffer. The measured transmission magnitude of the LTCC microwave-microfluidic sensor for various concentrations of dopamine in the buffer is presented in Figure 17. For both channels filled with the buffer solution, the S_21_ value was −50.7 dB and its value increased with the increase of the concentration of dopamine. As in the case of measurements for the water/ethanol mixtures, the S_21_ value increases were proportional to the rising concentrations of dopamine in the investigated sample, and a calculated calibration curve (Figure 18) was near linear (R^2^ = 0.994) with a slope equal to 0.023 dB/mM. The lowest measured concentration of dopamine was 0.1 mM. Below this value, the sensor’s response approached saturation.

## 5. Conclusions

The two types of microwave-microfluidic sensors were designed, fabricated and positively tested. Both devices were monolithic structures made with LTCC technology. Their principle of operation was based on the measurement of changes in resonant frequency and transmission magnitude, caused by changes in the composition of the fluid in the test microchannel. The resonator-based and coupler-based sensors were designed to operate at 2.5 GHz and 5 GHz, respectively. These two radio bands are very popular in telecommunication (e.g., WiFi, ZigBee). Thus, electronic components operating at these frequencies are inexpensive and widely available.

The presented microwave-microfluidic systems were designed according to results of numerical simulations. The calculations were made for resonator and coupler-based sensors filled with water. Fabricated LTCC sensors were tested for two types of test fluids—a water/ethanol mixture and various concentrations of dopamine in a buffer solution. They were able to detect differences in the composition of test fluids of different permittivity. The correlation between the change in resonant frequency (for the resonator-based sensor) and transmission magnitude (for the coupler-based sensor) and relative permittivity of the investigated test fluids were used for the direct determination of the analyte. The results of measurements were with a good accordance with performed simulations. For resonator-based sensor, the measured resonant frequency was 2.515 GHz and S_11_ = 24.4 dB. The difference between simulated and measured resonance frequency and amplitude of S_11_ were equal to 5 MHz and 0.6 dB, respectively. For the coupler-based sensor, the simulated and measured minimum of S_21_ were equal to −57.3 dB and −65.8 dB, respectively. The differences between the simulation and measurements were probably caused by differences between modeled and fabricated microwave components, e.g., the thickness of the silver conductor, substrate permittivity, and inaccuracy of the dimensions of the microstrip lines. The performed experiments have shown that the presented LTCC microwave-microfluidic sensors are sensitive to permittivity ranging from 25.3 to 80.1.

The measurements made for dopamine have shown that concentrations as small as 0.1 mM can only be detected using the coupler-based microwave-microfluidic sensor made with LTCC technology. According to the literature [30], the presented device can find practical applications in medicine and/or analytical chemistry. However, additional measurements for test fluids containing dopamine and interfering substances (e.g., ascorbic acid) should be performed.

Moreover, the presented LTCC-based sensors are passive, which means that they are scalable to higher frequencies. Therefore, they can be easily adapted to various microfluidic applications (e.g., the continuous monitoring of biomolecules). Both of the presented microwave-microfluidic systems can work as stand-alone devices or as part of more complex lab-on-chip or micro-total analysis systems (µTAS). 

Among other technologies that are used in the fabrication of microwave-microfluidic systems, LTCC technology can be a very interesting alternative. Inherent features of the LTCC (very good high-frequency dielectric properties, chemical resistance, biological inertness, high temperature stability, ease of micromechanical processing, the possibility of integrating different fluidic and electrical components) make it a promising material for the development of monolithic microwave-microfluidic systems for analytical chemistry, medicine or environmental monitoring.

## Figures and Tables

**Table 1:** Parameters of water and low temperature co-fired ceramic (LTCC) materials used in simulations.

Parameter	Water	LTCC
Conductivity (S/m)	5.5 × 10^−6^	10^−12^
Relative permittivity, *ε_r_*	80.1	7.8
